# Life Cycle Assessment of Impacted Tooth Surgery Under Different Clinical Scenarios

**DOI:** 10.3390/dj14070441

**Published:** 2026-07-15

**Authors:** Szidonia-Krisztina Veress, Adina Simona Coșarcă, Bálint Botond Bögözi, Bernadette Kerekes-Máthé, Melinda Székely

**Affiliations:** 1 Doctoral School of Medicine and Pharmacy, Instituția Organizatoare de Studii Universitare de Doctorat, George Emil Palade University of Medicine, Pharmacy, Science, and Technology of Targu Mures, 540142 Targu Mures, Romania; szidonia-krisztina.veress@umfst.ro; 2 Department of Oral and Maxilo-Facial Surgery, Faculty of Dentistry, George Emil Palade University of Medicine, Pharmacy, Science, and Technology of Targu Mures, 38 Gheorghe Marinescu Str., 540142 Targu Mures, Romania; balint.bogozi@umfst.ro; 3 Department of Teeth and Dental Arches Morphology, Faculty of Dentistry, George Emil Palade University of Medicine, Pharmacy, Science, and Technology of Targu Mures, 38 Gheorghe Marinescu Str., 540142 Targu Mures, Romaniamelinda.szekely@umfst.ro (M.S.)

**Keywords:** sustainable development, ecology, wisdom teeth, impacted teeth, tooth extraction, oral surgery, carbon footprint, disability adjusted life years

## Abstract

**Background/Objectives**: Dental procedures are generally not considered environmentally sustainable; however, there is a growing emphasis on the implementation of environmentally conscious practices in dentistry. Despite this shift, the environmental impact of oral surgical interventions remains insufficiently investigated. The odontectomy of impacted teeth represents the most frequently performed procedure in oral surgery. The aim of this study was to conduct a cradle-to-grave life cycle assessment of the odontectomy of submucosal and intraosseous impacted teeth. **Methods**: To perform the life cycle assessment, a life cycle inventory was compiled based on the observation and measurement of odontectomy procedures carried out at the dental clinic of George Emil Palade University of Medicine, Pharmacy, Science, and Technology of Târgu Mureș, Romania. The analysis was carried out using the OpenLCA 2.5 software and the ReCiPe 2016 v1.03 midpoint and endpoint impact categories. **Results**: Odontectomy of intraosseous impacted teeth had higher environmental impact values than submucosal procedures across all midpoint categories examined. The climate change (GWP) results were 1.43 kg CO_2_-eq (submucosal) and 1.56 kg CO_2_-eq (intraosseous) in the average-case scenario, while in the worst-case scenario these values increased to 2.21 and 2.35 kg CO_2_-eq, respectively. The overall DALY value was also higher in the intraosseous group. Single-use materials contributed the most to the majority of impact categories, while the contribution of reusable instruments was also noticeably higher in intraosseous procedures. **Conclusions**: A comparison of the two surgical procedures indicates that the environmental burden is primarily associated with the complexity of the procedure and the associated equipment requirements. Integrating sustainability considerations into clinical protocols could potentially yield significant environmental benefits in oral surgery.

## 1. Introduction

Climate change is the most significant health threat of the 21st century and places a significant burden on health care [1,2,3,4]. However, healthcare paradoxically contributes to environmental pollution and climate change, thereby indirectly harming health [5]. Healthcare emits significant amounts of greenhouse gases, accounting for 3–8% of the total carbon footprint of developed countries [6,7,8]. Environmental sustainability has therefore become a pressing global priority within the healthcare sector [9].

Dental care also has a significant environmental impact, primarily due to the high consumption of materials and energy, as well as the generation of infectious waste [10,11]. In its current form, dental care is considered unsustainable [10,12]. Therefore, a transition toward more environmentally sustainable dental practices is essential [13]. The World Dental Federation has also highlighted the importance of adopting environmentally responsible approaches to dental practice and actively encourages further research in this area [13,14]. Eco-friendly dentistry represents an emerging, holistic field that promotes environmental sustainability in dental care through prevention, minimally invasive interventions, efficient resource utilization, and waste reduction [15,16].

Preliminary evidence from questionnaire studies points to a possible willingness among dentists to engage in more environmentally sustainable practices; however, increasing their knowledge in this area appears to be necessary [17,18,19].

There is currently no guideline for sustainable dentistry [12]. A standardized method is needed to measure and evaluate environmental impacts, both to identify environmentally sustainable solutions and to monitor the sustainability of dental practices following the implementation of such measures [20]. Life Cycle Assessment (LCA) is a widely used method for evaluating environmental impacts and provides a robust methodological framework. It can assess the environmental impacts of a product or service using a cradle-to-gate approach, from raw material extraction through manufacturing and use phases to waste management [21,22,23]. LCA covers both midpoint and endpoint impact categories [24].

HealthcareLCA is an online database that compiles LCA results related to healthcare activities [25]. Although LCA is increasingly applied in oral healthcare, data on oral surgical interventions remain limited, and most studies report only midpoint results, while endpoint indicators such as disability-adjusted life years (DALY) are often lacking [26,27]. DALYs quantify the burden of disease as the years of healthy life lost due to premature mortality and time lived with illness or disability [28].

There is increasing interest in environmental awareness in dentistry; however, studies examining the environmental impacts of oral surgery remain limited. The most commonly performed oral surgical procedure is the odontectomy of impacted wisdom teeth [29,30,31,32]. Impacted mandibular wisdom teeth have a reported incidence ranging from 16.7% to 73.5% [30,31,32]. Impacted maxillary canines represent the second-most common type of impacted teeth, and odontectomy is the most frequently applied treatment for these cases [33]. Impacted teeth are defined as teeth that fail to erupt within the expected timeframe because of obstruction by bone, adjacent teeth, soft tissues or in case of eruption defects. Their removal is important because they may lead to several complications, including pericoronitis and the development of odontogenic cysts or tumors [30,32]. Odontectomy has several indications, including recurrent pericoronitis, orthodontic purposes, caries, and the prevention or treatment of tumoral complications [32].

The aim of this study was to conduct a life cycle assessment of the odontectomy of submucosal and intraosseous impacted teeth performed in a standard clinical setting at the George Emil Palade University of Medicine, Pharmacy, Science, and Technology of Târgu Mureș, Romania. To enhance interpretability and support decision-making, a sensitivity analysis was also performed. In addition to the baseline (average) case, ideal and worst-case scenarios were modeled. Environmental impacts were assessed using both midpoint categories and endpoint indicators. The latter are less commonly reported in literature. To the best of the authors’ knowledge, this is the first study to evaluate the life cycle environmental impacts of odontectomy of submucosally and intraosseous impacted teeth using this comprehensive approach.

## 2. Materials and Methods

The study was approved by the Ethics Committee of the George Emil Palade University of Medicine, Pharmacy, Science, and Technology of Târgu Mureș, Romania (approval number: 3363/approval date: 7 October 2024).

Life cycle assessment (LCA) is the gold-standard method for examining the sustainability of products or processes, and it is also used to assess the environmental impacts of healthcare activities.

The International Organization for Standardization (ISO) 14040:2006 standard and its 2020 amendment define the methodology for life cycle assessment. Accordingly, it comprises four steps:

1. defining the objective and scope
2. analyzing the life cycle inventory
3. performing a life cycle impact assessment
4. interpreting the results

### 2.1. Objective and Scope

#### 2.1.1. Goal and Scope

The aim of this study is to perform a life cycle assessment of the most frequently performed procedures in dentoalveolar surgery, namely the odontectomy of submucosal and intraosseous impacted teeth. In order to perform the sensitivity analysis, in addition to the life cycle assessment of the average case, life cycle analyses of the procedures performed according to the ideal and worst-case scenarios were also conducted for both surgical procedures. The procedures were subdivided into separate stages (e.g., anesthesia, extraction, waste management) to allow more detailed modeling and to allow the recognition of hotspot categories.

#### 2.1.2. Functional Unit

The functional unit refers to the clearly defined product or service that serves as the reference point for the life cycle assessment. In this study, the life cycle assessment was conducted for two functional units. In the first case, the odontectomy of one submucosally impacted tooth was considered, while in the second case, the odontectomy of one intraosseous impacted tooth was examined. Both procedures were performed at the clinic of the Faculty of Dentistry of George Emil Palade University of Medicine, Pharmacy, Science, and Technology of Târgu Mureș, Romania.

#### 2.1.3. System Boundaries

The system boundaries encompass all processes required to deliver the defined functional unit, including the associated materials, instruments, energy inputs, and generated waste streams. A cradle-to-grave approach was applied, covering all stages necessary to perform the investigated service. In this study, the boundaries include the production-related supply chain of materials and instruments, as well as their transportation from the manufacturer to the distributor and from the distributor to the point of use, namely the dental clinic of George Emil Palade University of Medicine, Pharmacy, Science, and Technology of Târgu Mureș, Romania. They further comprise the energy consumption (electricity and water) during the intervention, as well as the materials, disinfectants, and energy required for the cleaning, disinfection, and sterilization of reusable instruments following treatment. In addition, the management of generated waste is considered, distinguishing between household and medical waste streams.

Patient and staff transportation were excluded from the system boundaries, as these are highly variable, difficult to quantify accurately, and challenging to allocate to a specific intervention. Consequently, not all contributory factors associated with the two examined procedures are included; rather, the defined system boundaries provide a consistent and transparent framework for assessing the environmental impacts of the individual treatments under standardized conditions. Furthermore, device maintenance is excluded from the system boundaries, as it occurs infrequently and its environmental impact per individual treatment is considered negligible.

**Figure 1:**
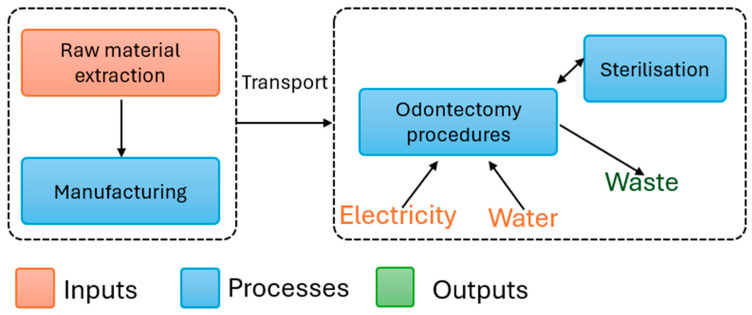
System boundaries.

The system boundaries are shown in Figure 1.

### 2.2. Analysis of the Life Cycle Inventory

The data collection was carried out at the dental and oral surgery clinic of the Faculty of Dentistry of George Emil Palade University of Medicine, Pharmacy, Science, and Technology of Târgu Mureș, Romania. The quantities of materials and devices required for the odontectomy of submucosal and intraosseous impacted teeth were determined based on empirical observations under ideal, average, and worst-case scenarios. The required materials and devices were disassembled into their components and weighed using a precision jewelry scale. Where possible, ten samples of each material or device were measured, and the average values were used. Information on the place of manufacture and the composition of the materials and instruments was obtained from packaging, product leaflets, and manufacturers’ websites. Energy consumption was determined based on the values provided in the user manuals of the devices used and was calculated proportionally according to the duration of use. The Life Cycle Inventory is provided in detail in Supplementary Material File S1 (Tables S1–S5).

The procedures were subdivided into the following stages: hand hygiene, preparation, anesthesia, surgical exploration and osteotomy, tooth extraction, curettage, alveolar bone regularization, hemostasis, disinfection, sterilization, and waste management. Each procedural step was associated with its corresponding material and energy inputs.

For the purposes of the calculations, a set of underlying assumptions was established to ensure consistency and comparability of the results:

1. The interventions were performed by one clinician with the assistance of one dental assistant.
2. The materials and instruments used were purchased monthly from a regional dental supplier, with transportation already included in the environmental impact of the respective products.
3. The pharmaceutical products were obtained from the nearest pharmacy, which is within walking distance; therefore, no additional transportation-related impacts were considered.
4. The materials and instruments were transported from their place of manufacture to the supplier via the shortest feasible routes, using a combination of sea and rail transport.
5. Disposable instruments were discarded after use, whereas the environmental impacts of reusable instruments were allocated across their assumed lifetime (500 sterilization cycles for hand instruments and 50 sterilization cycles for burs).
6. The duration of the interventions was assumed to be 1 h, corresponding to the operation time of the dental unit; however, the use duration of the physiodispenser and, where applicable, the turbine varies depending on the complexity of the procedure.
7. As the interventions involve exposure of the bone surface, both the operator and the assistant performed surgical hand antisepsis using Betadine prior to the procedure.
8. During the preparation phase, the surgical field was established under sterile conditions (sterile drapes and sterile gloves).
9. If required, bone removal was performed under irrigation with sterile saline.
10. In complex cases requiring tooth sectioning, this was carried out using a turbine and a diamond bur.
11. Following the intervention, the wound was closed using sutures.
12. After each procedure, the dental unit was disinfected using Mikrozid.
13. Disposable materials that have come into contact with patient body fluids were disposed of as infectious waste, whereas packaging materials were discarded as municipal (household) waste.
14. Reusable instruments were disinfected in a Gigasept solution, subsequently individually packaged, and sterilized in an autoclave.

### 2.3. Assessing the Life Cycle Impact

To assess the environmental impacts of the odontectomy of submucosally and intraosseous impacted teeth, a life cycle assessment was conducted using the OpenLCA software with the merged BAFU-2025_LCI DB_17Dec25, bioenergiedat_18, needs_18, OzLCI2019, USDA_1901009 and worldsteel_2020 databases. The life cycle impact assessment was performed using the ReCiPe 2016 v1.03 methodology, covering 18 midpoint impact categories as well as the endpoint category expressed in disability-adjusted life years (DALYs). The assessment was carried out for the average case, as well as for ideal and worst-case scenarios, in order to enable sensitivity analysis.

Based on the results, the parameters exerting the greatest influence on the outcomes were identified, and a lognormal uncertainty distribution with a factor of 1.3 was assigned to these variables. Uncertainty analysis was subsequently performed using 1000 Monte Carlo simulations.

## 3. Results

This section presents the results of the life cycle assessment for the two functional units examined: the odontectomy of submucosal and intraosseous impacted teeth.

Supplementary Material File S2 contains the results of the sensitivity and uncertainty analysis.

Table 1 and Table 2 present the results in the midpoint (H) impact categories for the odontectomy of submucosal and intraosseous impacted teeth under ideal, average, and worst-case scenarios.

**Figure 2:**
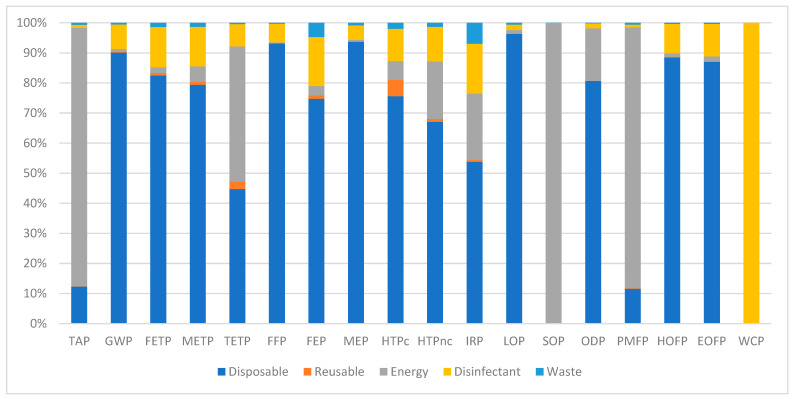
Breakdown of the relative contributions of individual factors to environmental impacts across 18 impact categories for the average scenario of submucosal impacted tooth odontectomy.

**Figure 3:**
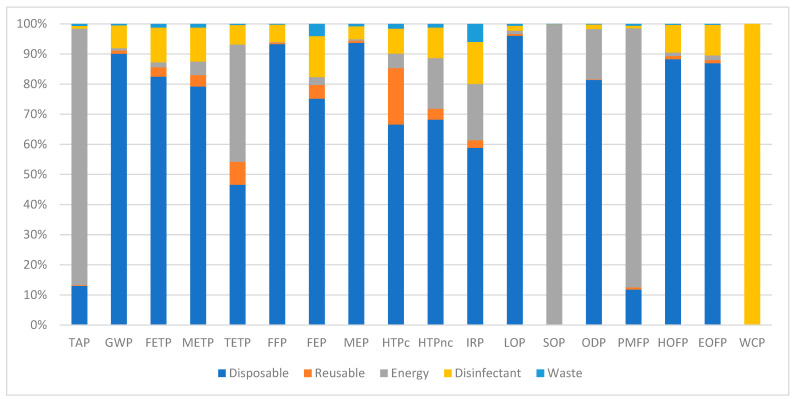
Breakdown of the relative contributions of individual factors to environmental impacts across 18 impact categories for the average scenario of intraossesous impacted tooth odontectomy.

Figure 2 and Figure 3 illustrate the relative contributions of individual factors (disposable and reusable materials and instruments, energy consumption, disinfectants and waste management) across 18 impact categories for the two procedures under average conditions. In most impact categories, single-use materials represented the largest contribution for both procedures, although the odontectomy of intraosseous impacted teeth also required the use of multiple reusable instruments.

To perform the uncertainty analysis, a lognormal distribution of uncertainty was assigned to the single-use materials. The results of the Monte Carlo simulations are presented in Supplementary Material File S2.

**Figure 4:**
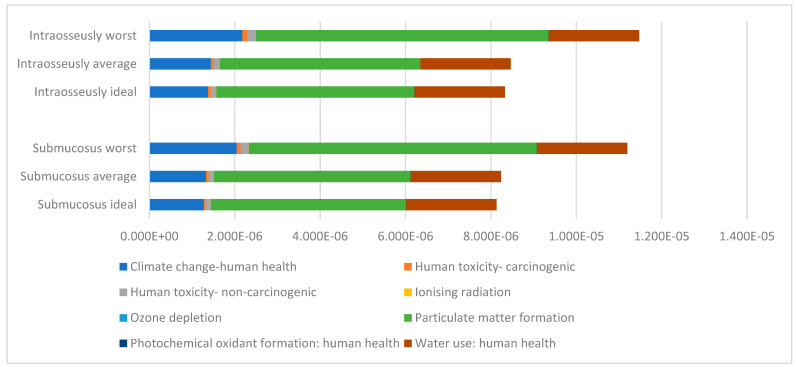
Contribution of impact categories to disability-adjusted life years (DALY) for the odontectomy of submucosally and intraosseous impacted teeth under ideal, average, and worst-case scenarios.

Table 3 summarizes the corresponding DALY values within the endpoint impact category for the odontectomy of intraosseous and submucosal impacted teeth across ideal, average, and worst-case conditions. Figure 4 illustrates the contribution of individual impact categories to the DALY results for the two procedures under the three scenarios examined.

## 4. Discussion

Healthcare contributes substantially to environmental pollution, highlighting the urgent need to adopt more environmentally sustainable approaches in the delivery of healthcare interventions [8]. Several organizations, including the World Health Organization and the World Dental Federation, have emphasized the importance of integrating environmental sustainability into healthcare practices, including dentistry [3,13,34,35]. Achieving this transition requires the systematic assessment of environmental impacts. To support reliable and comparable evaluations, measurement methods should be standardized [20,36]. Life cycle assessment (LCA) is an internationally standardized methodology that enables the quantification of environmental degradation associated with a given product or service throughout its entire life cycle [37].

The results demonstrate that both submucosal and intraosseous odontectomy are associated with significant environmental impacts, which generally increased progressively from the ideal-case to the worst-case scenario across most of the impact categories examined. Intraosseous odontectomy consistently exhibited higher environmental impact values, likely due to the greater complexity of the surgical technique, longer procedure duration, and increased use of instruments and materials. The most pronounced impacts were observed primarily in the categories of global warming potential (GWP), ecotoxicity, and fossil energy consumption. The DALY-based assessment further indicated that, among the impacts on human health, particulate matter formation and water consumption contributed most substantially to the overall health damage burden. Overall, the findings suggest that increasing surgical complexity is directly associated with greater environmental and human health impacts.

Across all interventions and scenarios examined, single-use materials were the dominant contributors to environmental impacts in most midpoint impact categories, with substantially higher usage in the worst-case scenario. Their production, transportation, and disposal place a considerable burden on the environment. The 4R principle has been introduced to promote environmentally sustainable dentistry. Reduce focuses on minimizing the use of single-use materials [38]. Reuse encourages replacing disposable products with sterilizable and reusable alternatives whenever feasible and maximizing their service life [15,36,39]. Recycle emphasizes proper waste segregation and recycling, with sterilization of infectious waste representing a potential additional strategy [8,36,38,40,41]. Finally, Rethink promotes staff education and the use of life cycle assessment (LCA) to identify environmental hotspots and support more sustainable treatment approaches [12,36,42].

Cross-infection during patient care remains a significant concern, contributing to the widespread use of single-use materials in dental practice [36]. The Centers for Disease Control and Prevention, in its Summary of Infection Prevention Practices in Dental Settings, which refers to the Guidelines for Infection Control in Dental Health-Care Settings—2003 [43], outlines several measures aimed at preventing the spread of nosocomial infections. These measures include the mandatory use of personal protective equipment, such as masks and gloves. However, the guidelines also identify sterilizable surgical textiles as a potential alternative in certain cases. Although the use of single-use materials is recommended to minimize the risk of infection transmission, the reuse of medical devices is permitted where feasible, provided that such practices comply with the manufacturer’s instructions.

The second-most significant contributor to environmental impact was energy consumption, primarily associated with the sterilization phase. This can largely be attributed to the high energy demand of autoclaves, despite their relatively short operating cycles [1]. The operation of a dental practice is inherently resource-intensive, requiring substantial amounts of electricity and water [36,44]. Optimizing energy consumption could therefore substantially reduce the environmental burden of dental care. Potential strategies include the use of energy-efficient equipment, on-site electricity generation, and the adoption of renewable energy sources, such as solar panels or wind turbines. In addition, relatively simple measures—such as operating the autoclave only when fully loaded and promoting responsible water use—may also contribute to improved environmental sustainability [8,26,36,45].

Disinfectants contributed substantially to environmental impacts across nearly all impact categories examined. Although sterilizable instruments represent a more environmentally sustainable alternative to single-use devices, their use requires disinfection procedures that often rely on substances toxic to aquatic ecosystems and living organisms [13,45,46]. Therefore, the adoption of alternative disinfectants that ensure effective sterilization while exerting lower environmental impacts would be highly desirable [47,48]. One such example is peracetic acid, which is not only sufficiently effective as a disinfectant but also degrades in water without forming harmful by-products [49].

DALYs are a health indicator representing the number of healthy life years lost due to premature mortality and morbidity [50]. Within life cycle assessment (LCA), DALYs can also be calculated as part of the ReCiPe Endpoint impact category [24]. Based on the DALY (disability-adjusted life year) results of the present study, both intervention types—treatment of submucosal and intraosseous impacted teeth—are associated with an extremely low health burden, with total values in the range of 10^−6^–10^−5^ DALYs, which is considered practically negligible at the population level. Among the contribution categories, particulate matter formation and climate change-related impacts were the dominant contributors, whereas the contributions of ionizing radiation and ozone depletion were marginal. In the worst-case scenarios, an increase was observed across all categories, reflecting the effects of more resource-intensive conditions. Overall, the difference between the two procedures was small but consistent, with intraosseous treatments resulting in a slightly higher DALY burden.

The most commonly performed oral surgical procedure is the odontectomy third molars [29,31]. In certain cases, coronectomy (partial odontectomy) is performed, in which only the crown of the tooth is removed, primarily to preserve the inferior alveolar nerve [29]. Such procedures may involve additional energy consumption due to tooth sectioning (worst-case scenario); however, if this is not accompanied by increased use of disposable materials, the additional environmental burden may remain limited. Nevertheless, a second surgical intervention may occasionally be required following coronectomy [31], potentially resulting in a substantially greater environmental impact compared to odontectomy completed in a single session. Accurate preoperative assessment is essential. This often involves cone-beam computed tomography (CBCT), as well as the use of predictive indices, such as the Pederson or Pernambuco index, which can help estimate procedural difficulty, anticipate resource requirements, and optimize the preparation of materials [31,32]. Although preoperative radiographic imaging and CBCT contribute to environmental burden, they are indispensable for safe and effective treatment planning. Importantly, digital radiology represents a considerably more environmentally sustainable alternative to conventional radiographic methods [1,36,38]. The second-most frequently impacted tooth is the maxillary canine, for which surgical intervention is also commonly required. These procedures are often more complex, involving both surgical exposure and orthodontic traction of the tooth [33]. Such interventions may further increase environmental impacts; however, these aspects were not included in any of the scenarios evaluated in the present study.

To date, no other study has quantitatively assessed the sustainability and environmental impact of odontectomy. The study Carbon Modeling within Dentistry: Towards a Sustainable Future (2018) estimated the carbon footprint of tooth extraction at 8.6 kg CO_2_e, although the extraction technique was not specified. Patient and staff travel were included and represented a major source of emissions [47]. Another cradle-to-grave life cycle assessment reported a footprint of 13.75 kg CO_2_eq for tooth extraction when separate visits were required for consent and treatment. Using digital consent and reducing the process to a single visit lowered emissions to 8.79 kg CO_2_eq. In both scenarios, travel accounted for the majority of emissions (74.1% and 61.6%, respectively). Differences between studies may also be related to variations in methodology and the use of the ecoinvent-based LCA model [51].

Based on the sensitivity analysis (results available in Supplementary Material File S2), the majority of environmental impacts associated with both types of odontectomy originated primarily from the preparation, sterilization, and, in certain cases, the extraction phases of the procedures. In the average- and worst-case scenarios, the preparation phase particularly dominated several impact categories, including GWP, FFP, and HOFP, suggesting that single-use materials represent a major source of environmental burden. Sterilization contributed especially prominently to the TAP, PMFP, SOP, and LOP categories, accounting in several instances for more than 50–90% of the total impact. This was mainly attributable to the high energy demand associated with sterilization processes. During intraosseous odontectomy, the contribution of exploration/drilling and bone-edge smoothing became more substantial, reflecting the greater complexity of the surgical technique and its increased energy requirements. In contrast, the contributions of anesthesia, hand hygiene, and waste management remained relatively minor across most impact categories. Overall, the findings indicate that reducing the environmental burden of odontectomy procedures could primarily be achieved through the optimization of single-use material consumption and sterilization practices.

Based on the uncertainty analysis (results available in Supplementary Material File S2), the results can generally be considered stable and reproducible; however, substantial variability was observed in certain impact categories. For most indicators, such as TAP, PMFP, and ODP, the low standard deviations and narrow 5th–95th percentile ranges indicate relatively low sensitivity to input parameters, suggesting that these results can be interpreted with a high degree of confidence. In contrast, the toxicity-related categories—particularly FETP, METP, HTPnc, and IRP—exhibited considerably higher standard deviations and wider minimum–maximum ranges, reflecting greater uncertainty. This suggests that the results in these categories are strongly influenced by a limited number of dominant processes or by uncertainties in the background data. In the worst-case scenarios, the level of uncertainty increased further, as demonstrated by higher standard deviations and extreme maximum values, especially in the case of intraosseous odontectomy. Nevertheless, the median values were, in most cases, close to the mean values, indicating that the distributions were not markedly skewed and that the majority of the results remained within a relatively concentrated range. Overall, the uncertainty analysis demonstrates that the principal environmental trends can be identified reliably; however, the interpretation of certain toxicity-related categories requires increased caution due to their higher associated uncertainty.

Reducing the use of single-use materials and prioritizing sterilizable instruments, together with optimizing preparation and sterilization processes, as well as ensuring appropriate and selective waste collection, may all contribute to decreasing the environmental impact of odontectomy procedures. In addition, increasing staff awareness and implementing a green supply chain are also essential [12,44,52]. Improving the environmental sustainability of odontectomy and other oral surgical interventions requires engagement from manufacturers and all stakeholders throughout the supply chain [28,44,53], with staff education and continued research into more sustainable alternatives [7,34,54].

### Limitations of the Study

A limitation of this study is that the databases used did not always provide country-specific conversion factors; therefore, global average values were applied in certain cases. In addition, some of the database values were based on older datasets that may have changed over time. Furthermore, part of the data relied on assumptions; however, these assumptions were grounded in careful observation of the clinical interventions performed at the study site. Another limitation is that the measurements were conducted in a single clinical center. Therefore, broader interpretation and generalizability of the findings would require comparison with other centers using different materials and potentially different technologies. A further limitation of the study is that the system boundaries included only the processes directly related to the performance of the procedure itself. However, odontectomy involves several additional factors that were not considered in the analysis, including preoperative radiographic examinations, clinic maintenance, as well as transportation associated with both patients and healthcare staff.

## 5. Conclusions

Among the two evaluated procedures, the odontectomy of intraosseous impacted teeth consistently showed higher environmental impacts across all impact categories compared with the submucosal approach. The results indicate that single-use materials were the dominant contributor to the overall environmental burdens in both procedures, representing the primary hotspot driving most impact categories. According to the DALY results, the main human health-related impacts were primarily associated with climate change and particulate matter formation. The worst-case scenarios further amplified environmental burdens across all categories, highlighting the influence of variability in clinical practice and material use. Overall, the findings suggest that targeted reduction in single-use materials, without compromising patient safety, is key to improving the environmental sustainability of oral surgical care.

## Figures and Tables

**Table 1:** Life cycle impact assessment results (18 midpoint (H) categories) for submucosally impacted teeth odontectomy across three scenarios.

Impact Category	Reference Unit	Ideal Case	Average Case	Worst Case
**Acidification: terrestrial (TAP)**	kg SO_2_-Eq	2.16 × 10^−2^	2.18 × 10^−2^	3.19 × 10^−2^
**Climate change (GWP)**	kg CO_2_-Eq	1.37	1.43	2.21
**Ecotoxicity: freshwater (FETP)**	kg 1,4-DCB-Eq	1.05 × 10^−2^	1.13 × 10^−2^	1.76 × 10^−2^
**Ecotoxicity: marine (METP)**	kg 1,4-DCB-Eq	1.54 × 10^−2^	1.66 × 10^−2^	2.58 × 10^−2^
**Ecotoxicity: terrestrial (TETP)**	kg 1,4-DCB-Eq	2.18	2.28	3.45
**Energy resources: non-renewable, fossil (FFP)**	kg oil-Eq	7.19 × 10^−1^	7.38 × 10^−1^	1.12
**Eutrophication: freshwater (FEP)**	kg P-Eq	1.02 × 10^−4^	1.14 × 10^−4^	1.73 × 10^−4^
**Eutrophication: marine (MEP)**	kg N-Eq	4.26 × 10^−5^	5.50 × 10^−5^	8.47 × 10^−5^
**Human toxicity: carcinogenic (HTPc)**	kg 1,4-DCB-Eq	1.46 × 10^−2^	1.60 × 10^−2^	2.67 × 10^−2^
**Human toxicity: non-carcinogenic (HTPnc)**	kg 1,4-DCB-Eq	5.10 × 10^−1^	5.51 × 10^−1^	8.44 × 10^−1^
**Ionizing radiation (IRP)**	kBq Co-60-Eq	1.03 × 10^−1^	1.15 × 10^−1^	1.72 × 10^−1^
**Land use (LOP)**	m^2^*a crop-Eq	3.87 × 10^−2^	4.74 × 10^−2^	7.20 × 10^−2^
**Material resources: metals/minerals (SOP)**	kg Cu-Eq	1.92	1.93	2.79
**Ozone depletion (ODP)**	kg CFC-11-Eq	1.51 × 10^−6^	1.67 × 10^−6^	3.00 × 10^−6^
**Particulate matter formation (PMFP)**	kg PM2.5-Eq	7.28 × 10^−3^	7.34 × 10^−3^	1.07 × 10^−2^
**Photochemical oxidant formation: human health (HOFP)**	kg NOx-Eq	3.04 × 10^−3^	3.13 × 10^−3^	4.78 × 10^−3^
**Photochemical oxidant formation: terrestrial ecosystems (EOFP)**	kg NOx-Eq	3.23 × 10^−3^	3.32 × 10^−3^	5.05 × 10^−3^
**Water use (WCP)**	m^3^	9.53 × 10^−1^	9.53 × 10^−1^	9.53 × 10^−1^

**Table 2:** Life cycle impact assessment results (18 midpoint (H) categories) for intraossesus impacted teeth odontectomy across three scenarios.

Impact Category	Reference Unit	Ideal Case	Average Case	Worst Case
**Acidification: terrestrial (TAP)**	kg SO_2_-Eq	2.18 × 10^−2^	2.21 × 10^−2^	3.22 × 10^−2^
**Climate change (GWP)**	kg CO_2_-Eq	1.48	1.56	2.35
**Ecotoxicity: freshwater (FETP)**	kg 1,4-DCB-Eq	1.20 × 10^−2^	1.32 × 10^−2^	1.98 × 10^−2^
**Ecotoxicity: marine (METP)**	kg 1,4-DCB-Eq	1.76 × 10^−2^	1.93 × 10^−2^	2.90 × 10^−2^
**Ecotoxicity: terrestrial (TETP)**	kg 1,4-DCB-Eq	2.52	2.64	3.94
**Energy resources: non-renewable, fossil (FFP)**	kg oil-Eq	7.69 × 10^−1^	7.98 × 10^−1^	1.19
**Eutrophication: freshwater (FEP)**	kg P-Eq	1.20 × 10^−4^	1.36 × 10^−4^	2.01 × 10^−4^
**Eutrophication: marine (MEP)**	kg N-Eq	4.86 × 10^−5^	6.14 × 10^−5^	9.15 × 10^−5^
**Human toxicity: carcinogenic (HTPc)**	kg 1,4-DCB-Eq	1.90 × 10^−2^	2.07 × 10^−2^	3.32 × 10^−2^
**Human toxicity: non-carcinogenic (HTPnc)**	kg 1,4-DCB-Eq	5.73 × 10^−1^	6.26 × 10^−1^	9.35 × 10^−1^
**Ionizing radiation (IRP)**	kBq Co-60-Eq	1.19 × 10^−1^	1.37 × 10^−1^	1.98 × 10^−1^
**Land use (LOP)**	m^2^*a crop-Eq	4.21 × 10^−2^	5.10 × 10^−2^	7.60 × 10^−2^
**Material resources: metals/minerals (SOP)**	kg Cu-Eq	1.93	1.93	2.80
**Ozone depletion (ODP)**	kg CFC-11-Eq	1.59 × 10^−6^	1.75 × 10^−6^	3.09 × 10^−6^
**Particulate matter formation (PMFP)**	kg PM2.5-Eq	7.39 × 10^−3^	7.47 × 10^−3^	1.09 × 10^−2^
**Photochemical oxidant formation: human health (HOFP)**	kg NOx-Eq	3.23 × 10^−3^	3.35 × 10^−3^	5.02 × 10^−3^
**Photochemical oxidant formation: terrestrial ecosystems (EOFP)**	kg NOx-Eq	3.43 × 10^−3^	3.55 × 10^−3^	5.31 × 10^−3^
**Water use (WCP)**	m^3^	9.53 × 10^−1^	9.53 × 10^−1^	9.53 × 10^−1^

**Table 3:** Disability-adjusted life years (DALY) results within endpoint (H) impact categories for the odontectomy of submucosally and intraosseous impacted teeth under ideal, average, and worst-case scenarios.

DALYs	Submucosal Impacted Teeth			Intraosseous Impacted Teeth		
	Ideal Case	Average Case	Worst Case	Ideal Case	Average Case	Worst Case
**Climate change: human health**	1.27 × 10^−6^	1.32 × 10^−6^	2.04 × 10^−6^	1.37 × 10^−6^	1.44 × 10^−6^	2.17 × 10^−6^
**Human toxicity: carcinogenic**	4.85 × 10^−8^	5.31 × 10^−8^	8.87 × 10^−8^	6.29 × 10^−8^	6.88 × 10^−8^	1.10 × 10^−7^
**Human toxicity: non-carcinogenic**	1.16 × 10^−7^	1.25 × 10^−7^	1.92 × 10^−7^	1.30 × 10^−7^	1.42 × 10^−7^	2.13 × 10^−7^
**Ionizing** **radiation**	8.72 × 10^−10^	9.78 × 10^−10^	1.46 × 10^−9^	1.01 × 10^−9^	1.16 × 10^−9^	1.68 × 10^−9^
**Ozone depletion**	7.99 × 10^−10^	8.84 × 10^−10^	1.59 × 10^−9^	8.43 × 10^−10^	9.29 × 10^−10^	1.64 × 10^−9^
**Particulate matter formation**	4.57 × 10^−6^	4.61 × 10^−6^	6.74 × 10^−6^	4.63 × 10^−6^	4.68 × 10^−6^	6.84 × 10^−6^
**Photochemical oxidant formation: human health**	2.76 × 10^−9^	2.85 × 10^−9^	4.34 × 10^−9^	2.94 × 10^−9^	3.04 × 10^−9^	4.57 × 10^−9^
**Water use: human health**	2.11 × 10^−6^	2.11 × 10^−6^	2.11 × 10^−6^	2.11 × 10^−6^	2.11 × 10^−6^	2.11 × 10^−6^
**Total:**	8.12 × 10^−6^	8.23 × 10^−6^	1.11 × 10^−5^	8.32 × 10^−6^	8.46 × 10^−6^	1.14 × 10^−5^
**Total in days:**	0.00296	0.00300	0.00405	0.00303	0.00308	0.00416
**Total in hours:**	0.071	0.072	0.097	0.072	0.073	0.099

## Data Availability

Data presented is available upon request from the corresponding author.

## Supplementary Materials

The following supporting information can be downloaded at: https://www.mdpi.com/article/10.3390/dj14070441/s1, Supplementary Materials File S1: Life Cycle Inventory; Table S1: Life cycle inventory of used resuable instruments; Table S2: Life cycle inventory of used disposable materials; Table S3: Life cycle inventory of disinfectants; Table S4: Life cycle inventory of used energy; Table S5: Life cycle inventory of used resources; Supplementary Materials File S2: Results of Uncertainty and Sensitivity Analysis.

## Abbreviations

The following abbreviations are used in this manuscript:

LCA	Life Cycle Assessment
DALY	Disability Adjusted Life Years
CBCT	cone-beam computed tomography
ISO	International Organization for Standardization
