# Supplementary material for: Life Cycle Assessment of Impacted Tooth Surgery Under Different Clinical Scenarios
Source: Dent J (Basel). 2026 Jul 15;14(7):441. doi: 10.3390/dj14070441 (PMC13409643; doi:10.3390/dj14070441)
Supplement: Supplementary file 1 [file dentistry-14-00441-s001.zip › Supplementary material 1.pdf]

Supplementary Material 1: Life Cycle Inventory

Table S1: Life cycle inventory of used resuable instruments

Instrument	Composition	Mass (g)	Lifespan	Country of origin
**dental mirror**	stainless steel+ glass	22,1	500	Pakistan
**dental tweezers**	stainless steel	22,7	500	Pakistan
**dental probe**	stainless steel	18,8	500	Pakistan
**surgical aspirator**	polypropylene	3,087	50	Italy
**atraumatic syringe**	stainless steel	62	500	Pakistan
**scalpel handle**	stainless steel	22,5	500	Pakistan
**surgical retractor**	stainless steel	20,8	500	Japan
**periosteal elevator**	stainless steel	30,9	500	Pakistan
**straight elevator**	stainless steel	57,6	500	Pakistan
**luxator**	stainless steel+ plastic	61	500	Sweden
**extraction forceps**	stainless steel	152,2	500	Pakistan
**Pean hemostatic forceps**	stainless steel	33,7	500	Japán
**surgical curette**	stainless steel	17,5	500	Pakistan
**bone rongeur**	stainless steel	95	500	Pakistan
**needle holder**	stainless steel	49	500	Pakistan
**surgical forceps**	stainless steel	22,4	500	Japan
**surgical scrissors**	stainless steel	21,5	500	Japan
**round bur**	tungsten carbide	1,451	50	Austria
**diamond bur**	stainless steel + diamond grit	0,269	50	Israel
**small packaging (burs)**	paper + nylon	1,016	1	Finland
**big packaging (hand instruments)**	paper + nylon	2,456	1	Finland

Table S2: Life cycle inventory of used disposable materials

	Material		Individual packaging		Bulk packaging			Country of origin
	Composition	Mass (g)	Composition	Mass (g)	Composition	Mass (g)	Nr of items	
**surgical mask**	non woven material + metal + meltbown farbric	1,297			polyethylene	4,407	50	China
**dental napkin**	paper towel	1,012			polyethylene + cardboard	67,207	400	Romania
**disposable gloves**	nitrile	2,860			PAP 21	44,667	100	Malaysia
**sterile surgical gloves**	latex	21,583	paper + plastic	9,264	cardboard	137	50	Austria
**sterile drape**	SSMMS 35g non-woven fabric	170	paper + plastic	9,612			1	Turkey
**saliva ejector**	non-toxic PVC	4,167			polyethylene	4,054	100	Italy
**disposable cup**	bamboo fiber	2,808			polyethylene	2,096	50	Sweden
**atraumatic needle**	stainless steel+ polypropylene	0,213	polypropylene	1,502	PAP 21	29,461	100	Korea
**gauze swab**	cotton	0,748			polyethylene	0,963	32	Romania
**scalplel blade**	stainless steel	0,348	aluminium foil + cardboard	0,611	PAP 21	11,657	100	India
**10 mL syringe**	polypropylene	6,700	PAP 22 + polyethylene + polypropylene	1,383	PAP 21	118	100	China
**irrigation needle**	stainless steel + polypropylene	0,246						
**suture thread**	stainless steel + silk thread	0,064	aluminium foil + paper	3,065	cardboard + nylon	25,354	12	Greece
**articaine cartridge**	articaine solution		glass					
**saline solution**	saline solution	0,500	polyethylene	120				Romania

Table S3: Life cycle inventory of disinfectants

Disinfectant	Qantity
**Betadine**	30 g / surgical scrub
**Gigasept**	12 g Gigasept + 6988g water/ instrument disinfection procedure
**Mikrozid**	30 g / dental unit disinfection

Table S4: Life cycle inventory of used energy

Machine	Brand	Power (W)	Water (litres)
**Dental unit**	Dentsply Sirona Intego	100 Wh	
**Turbine**	Sinol AZL-4	0 Wh	50 ml/sec
**Physiodispenser**	NSK Surgic XT	48 Wh	
**Sealing device**	Melag Melaseal 100+	300 W	
**Autoclave**	Melag Euroklav 23 VS+	2300 Wh	700 ml/sterilization cycle
**Compressor**	Atlas Copco	1000 Wh	

Table S5: Life cycle inventory of used resources

		Submucosal odontectomy			Intraosseous odontectomy		
		Ideal case	Average case	Worst case	Ideal case	Average case	Worst case
**Hand washing**	Betadine	30 g	30 g	30 g	30 g	30 g	30 g
	tap water	20 kg	20 kg	20 kg	20 kg	20 kg	20 kg
**Preparation**	electricity	50 Wh	50 Wh	100 Wh	50 Wh	50 Wh	100 Wh
	surgical mask	2	2	2	2	2	2
	dental napkin	1	2	3	1	2	3
	disposable gloves	0	0	2	0	0	2
	sterile surgical gloves	2	2	3	2	2	3
	consultation set	0	1	2	0	1	2
	sterile drape	2	2	3	2	2	3
	saliva ejector	0	0	1	0	0	1
	surgical aspirator	1	1	1	1	1	1
	disposable cup	1	1	1	1	1	1
	tap water	360 g	360g	360g	360g	360g	360g
**Anesthesia**	articaine cartridge	1	2	2	1	2	2
	atraumatic needle	1	2	2	1	2	2
	atraumatic syringe	1	1	1	1	1	1
	gauze swab	0	0	1	0	0	1
**Exploration, drilling**	electricity	0	0	0	0,8 Wh	1,6 Wh	2,4 Wh
	scalpel handle	1	1	1	1	1	1
	scalpel blade	1	1	2	1	1	2
	periosteal elevator	1	1	1	1	1	1
	surgical retractor	1	1	2	1	1	2
	round bur	0	0	0	1	1	2
	10 mL syringe	0	0	0	1	1	2
	irrigation needle	0	0	0	1	1	2
	saline solution	0	0	0	30 ml	60 ml	90 ml
**Extraction**	electricity	0	0	0	0	0	334 kW
	straight elevator	1	1	1	1	1	1

	luxator	0	1	1	0	1	1
	extarction forceps	0	1	1	0	1	1
	Pean hemostatic forceps	0	0	1	0	0	1
	gauze swab	3	4	5	4	5	6
	diamond bur	0	0	1	0	0	1
	deionised water	0	0	0	0	0	1 kg
**Curettage**	surgical curette	1	1	1	1	1	1
	gauze swab	1	2	3	1	2	3
**Bone edge smoothing**	electricity	0	0	0	0,4 Wh	0,4 Wh	0,4 Wh
	bone rongeur	0	0	1	0	0	0
	surgical retractor	0	0	1	0	0	1
	round bur	0	0	0	0	0	1
	10 mL syringe	0	0	1	0	1	1
	irrigation needle	0	0	1	0	1	1
	saline solution	0	0	10 g	25 g	25 g	25 g
**Hemostasis, closure**	gauze swab	2	3	4	2	3	4
	needle holder	1	1	1	1	1	1
	surgical forceps	1	1	1	1	1	1
	surgical scrissors	1	1	1	1	1	1
	suture thread	1	1	2	1	1	2
**Disinfection**	disposable gloves	0	2	2	0	2	2
	Mikrozid	30 g	30 g	30 g	30 g	30 g	30 g
	dental napkin	1	1	1	1	1	1
**Sterilisation**	electricity	811,6 Wh	813,66 Wh	817,67 Wh	812,33 Wh	814,33 Wh	819 Wh
	Gigasept	12 ml	12 ml	12 ml	12 ml	12 ml	12 ml
	small sterile packaging	0	0	1	1	1	4
	large sterile packaging	10	13	18	10	13	17
	tap water	6988 g	8188 g	10238 g	7038 g	8238 g	9988 g
**Waste**	municipal waste	75,495 g	83,451 g	124,324 g	78,318 g	86,274 g	129,536 g
	infectious waste	396,63 g	405,819 g	620,324 g	404,324 g	413,759 g	634,964 g
