# Supplementary material for: Life Cycle Assessment of Impacted Tooth Surgery Under Different Clinical Scenarios
Source: Dent J (Basel). 2026 Jul 15;14(7):441. doi: 10.3390/dj14070441 (PMC13409643; doi:10.3390/dj14070441)
Supplement: Supplementary file 1 [file dentistry-14-00441-s001.zip › Supplementary material 2.pdf]

Supplementary material 2:

Sensitivity analysis:

1. Submucosal odontectomy – ideal case - Percentage contribution of the different procedural steps to the overall environmental impacts

Impact category	Reference unit	Results	Hand washing	Preparation	Anesthesia	Exploration, drilling	Extraction	Curettage	Bone edge smoothing	Hemostasis, closure	Disinfection	Sterilisation	Waste
**TAP**	kg SO2-Eq	2,16E-02	0,15%	14,49%	0,10%	0,11%	0,56%	0,19%	0,00%	0,78%	0,39%	82,54%	0,69%
**GWP**	kg CO2-Eq	1,37E+00	1,46%	73,96%	0,47%	1,47%	2,65%	0,88%	0,00%	7,20%	4,03%	7,36%	0,52%
**FETP**	kg 1,4-DCB-Eq	1,05E-02	1,76%	63,25%	0,61%	0,20%	5,82%	1,94%	0,00%	4,73%	7,47%	12,79%	1,43%
**METP**	kg 1,4-DCB-Eq	1,54E-02	1,71%	60,88%	0,63%	0,25%	5,48%	1,82%	0,00%	4,62%	7,30%	15,89%	1,42%
**TETP**	kg 1,4-DCB-Eq	2,18E+00	0,55%	32,90%	0,80%	1,37%	2,94%	0,97%	0,00%	5,81%	3,09%	51,15%	0,42%
**FFP**	kg oil-Eq	7,19E-01	0,50%	86,76%	0,47%	0,03%	1,09%	0,36%	0,00%	0,88%	4,14%	5,50%	0,27%
**FEP**	kg P-Eq	1,02E-04	2,90%	47,15%	0,66%	0,29%	8,95%	2,98%	0,00%	6,96%	9,16%	15,81%	5,14%
**MEP**	kg N-Eq	4,26E-05	1,56%	32,70%	0,19%	0,10%	27,83%	9,28%	0,00%	19,15%	2,23%	5,86%	1,10%
**HTPc**	kg 1,4-DCB-Eq	1,46E-02	1,64%	57,99%	1,36%	1,09%	6,24%	2,06%	0,00%	5,54%	5,87%	16,07%	2,14%
**HTPnc**	kg 1,4-DCB-Eq	5,10E-01	1,68%	49,17%	0,55%	0,26%	5,76%	1,92%	0,00%	4,76%	6,27%	28,22%	1,41%
**IRP**	kBq Co-60-Eq	1,03E-01	3,63%	23,66%	0,49%	0,19%	9,00%	3,00%	0,00%	7,19%	9,34%	35,95%	7,55%
**LOP**	m2*a crop-Eq	3,87E-02	1,03%	20,00%	0,20%	0,60%	1,93%	0,64%	0,00%	15,51%	5,67%	53,68%	0,74%
**SOP**	kg Cu-Eq	1,92E+00	0,11%	5,80%	0,03%	0,00%	0,00%	0,00%	0,00%	0,00%	0,00%	94,03%	0,03%
**ODP**	kg CFC-11-Eq	1,51E-06	0,26%	24,93%	0,18%	6,83%	10,01%	3,34%	0,00%	33,58%	0,63%	20,06%	0,18%
**PMFP**	kg PM2.5-Eq	7,28E-03	0,15%	14,34%	0,11%	0,12%	0,37%	0,12%	0,00%	0,66%	0,41%	83,12%	0,60%
**HOFP**	kg NOx-Eq	3,04E-03	1,89%	72,44%	0,41%	1,94%	1,48%	0,49%	0,00%	8,20%	4,64%	8,13%	0,38%
**EOFP**	kg NOx-Eq	3,23E-03	1,81%	71,51%	0,42%	1,83%	1,45%	0,48%	0,00%	7,78%	5,83%	8,52%	0,37%
**WCP**	m3	9,53E-01	99,98%	0,01%	0,00%	0,00%	0,00%	0,00%	0,00%	0,00%	0,00%	0,01%	0,00%

2. Submucosal odontectomy – average case - Percentage contribution of the different procedural steps to the overall environmental impacts

Impact category	Reference unit	Results	Hand washing	Preparation	Anesthesia	Exploration, drilling	Extraction	Curettage	Bone edge smoothing	Hemostasis, closure	Disinfection	Sterilisation	Waste
**TAP**	kg SO2-Eq	2,18E-02	0,15%	14,36%	0,19%	0,11%	0,74%	0,37%	0,00%	0,96%	0,39%	82,04%	0,69%
**GWP**	kg CO2-Eq	1,43E+00	1,40%	71,06%	0,86%	1,41%	3,41%	1,68%	0,00%	7,74%	3,86%	8,05%	0,53%
**FETP**	kg 1,4-DCB-Eq	1,13E-02	1,63%	58,67%	1,00%	0,19%	7,24%	3,54%	0,00%	6,12%	6,89%	13,35%	1,37%
**METP**	kg 1,4-DCB-Eq	1,66E-02	1,59%	56,69%	1,01%	0,24%	6,85%	3,33%	0,00%	5,92%	6,76%	16,26%	1,35%
**TETP**	kg 1,4-DCB-Eq	2,28E+00	0,52%	49,72%	1,06%	1,31%	4,00%	1,71%	0,00%	6,34%	2,95%	31,97%	0,42%
**FFP**	kg oil-Eq	7,38E-01	0,49%	84,56%	0,89%	0,03%	1,43%	0,70%	0,00%	1,20%	4,03%	6,39%	0,28%
**FEP**	kg P-Eq	1,14E-04	2,59%	42,46%	1,00%	0,26%	10,76%	5,27%	0,00%	8,82%	8,19%	15,91%	4,74%
**MEP**	kg N-Eq	5,50E-05	1,21%	25,46%	0,27%	0,08%	28,78%	14,38%	0,00%	22,03%	1,73%	5,20%	0,86%
**HTPc**	kg 1,4-DCB-Eq	1,60E-02	1,50%	53,98%	1,68%	1,00%	8,03%	3,53%	0,00%	6,71%	5,37%	16,19%	2,01%
**HTPnc**	kg 1,4-DCB-Eq	5,51E-01	1,56%	45,84%	0,88%	0,24%	7,20%	3,51%	0,00%	6,15%	5,81%	27,46%	1,35%
**IRP**	kBq Co-60-Eq	1,15E-01	3,24%	21,37%	0,77%	0,17%	10,75%	5,31%	0,00%	9,05%	8,32%	34,07%	6,95%
**LOP**	m2*a crop-Eq	4,74E-02	0,84%	20,12%	0,30%	0,49%	2,11%	1,04%	0,00%	13,18%	4,63%	56,67%	0,62%
**SOP**	kg Cu-Eq	1,93E+00	0,11%	5,79%	0,06%	0,00%	0,00%	0,00%	0,00%	0,00%	0,00%	94,02%	0,02%
**ODP**	kg CFC-11-Eq	1,67E-06	0,24%	22,59%	0,32%	6,18%	12,07%	6,03%	0,00%	33,38%	0,57%	18,44%	0,18%
**PMFP**	kg PM2.5-Eq	7,34E-03	0,14%	14,26%	0,19%	0,12%	0,51%	0,24%	0,00%	0,77%	0,41%	82,76%	0,60%
**HOFP**	kg NOx-Eq	3,13E-03	1,84%	70,46%	0,76%	1,89%	1,94%	0,94%	0,00%	8,42%	4,51%	8,87%	0,37%
**EOFP**	kg NOx-Eq	3,32E-03	1,76%	69,58%	0,77%	1,78%	1,90%	0,93%	0,00%	8,01%	5,66%	9,26%	0,35%
**WCP**	m3	9,53E-01	99,97%	0,01%	0,01%	0,00%	0,00%	0,00%	0,00%	0,01%	0,00%	0,00%	0,00%

3. Submucosal odontectomy – worst case - Percentage contribution of the different procedural steps to the overall environmental impacts

Impact category	Reference unit	Results	Hand washing	Preparation	Anesthesia	Exploration, drilling	Extraction	Curettage	Bone edge smoothing	Hemostasis, closure	Disinfection	Sterilisation	Waste
**TAP**	kg SO2-Eq	3,19E-02	0,10%	16,66%	0,25%	0,27%	23,42%	0,38%	0,15%	1,05%	0,40%	56,59%	0,73%
**GWP**	kg CO2-Eq	2,21E+00	0,91%	70,52%	1,10%	2,35%	2,97%	1,63%	1,32%	8,93%	3,38%	6,38%	0,51%
**FETP**	kg 1,4-DCB-Eq	1,76E-02	1,05%	60,82%	1,77%	1,30%	6,15%	3,41%	1,60%	5,51%	6,64%	10,41%	1,34%
**METP**	kg 1,4-DCB-Eq	2,58E-02	1,02%	58,92%	1,72%	1,28%	6,69%	3,21%	1,54%	5,37%	6,63%	12,30%	1,32%
**TETP**	kg 1,4-DCB-Eq	3,45E+00	0,35%	33,29%	1,22%	1,98%	15,41%	1,66%	0,69%	6,88%	4,13%	33,98%	0,41%
**FFP**	kg oil-Eq	1,12E+00	0,32%	84,92%	0,82%	0,26%	1,21%	0,69%	1,39%	1,11%	3,59%	5,41%	0,28%
**FEP**	kg P-Eq	1,73E-04	1,70%	44,95%	2,37%	1,95%	9,32%	5,17%	1,63%	7,98%	7,64%	12,55%	4,74%
**MEP**	kg N-Eq	8,47E-05	0,78%	25,42%	4,84%	4,76%	23,44%	14,00%	0,68%	19,25%	1,81%	4,15%	0,87%
**HTPc**	kg 1,4-DCB-Eq	2,67E-02	0,90%	59,74%	1,99%	1,77%	8,19%	3,10%	1,24%	5,35%	4,53%	11,34%	1,85%
**HTPnc**	kg 1,4-DCB-Eq	8,44E-01	1,02%	48,21%	1,71%	1,37%	10,54%	3,43%	1,23%	5,62%	6,05%	19,48%	1,34%
**IRP**	kBq Co-60-Eq	1,72E-01	2,17%	24,01%	2,29%	1,94%	14,10%	5,33%	1,39%	8,49%	7,86%	25,33%	7,09%
**LOP**	m2*a crop-Eq	7,20E-02	0,56%	17,52%	0,54%	0,98%	1,82%	1,03%	1,11%	16,67%	6,19%	52,98%	0,60%
**SOP**	kg Cu-Eq	2,79E+00	0,07%	7,99%	0,04%	0,00%	26,65%	0,00%	0,00%	0,00%	0,00%	65,22%	0,03%
**ODP**	kg CFC-11-Eq	3,00E-06	0,13%	26,96%	1,85%	8,52%	12,11%	5,01%	0,39%	33,66%	0,70%	10,52%	0,15%
**PMFP**	kg PM2.5-Eq	1,07E-02	0,10%	16,70%	0,21%	0,23%	23,39%	0,24%	0,16%	0,87%	0,43%	57,04%	0,63%
**HOFP**	kg NOx-Eq	4,78E-03	1,20%	69,74%	0,81%	2,76%	1,89%	0,93%	1,23%	10,40%	3,69%	6,99%	0,36%
**EOFP**	kg NOx-Eq	5,05E-03	1,16%	69,25%	0,81%	2,62%	1,99%	0,91%	1,22%	9,90%	4,52%	7,26%	0,36%
**WCP**	m3	9,53E-01	99,96%	0,01%	0,00%	0,00%	0,00%	0,00%	0,00%	0,01%	0,00%	0,02%	0,00%

4. Intraosseous odontectomy – ideal case - Percentage contribution of the different procedural steps to the overall environmental impacts

Impact category	Reference unit	Results	Hand washing	Preparation	Anesthesia	Exploration, drilling	Extraction	Curettage	Bone edge smoothing	Hemostasis, closure	Disinfection	Sterilisation	Waste
**TAP**	kg SO2-Eq	2,18E-02	0,15%	14,31%	0,10%	0,59%	0,73%	0,18%	0,42%	0,78%	0,39%	81,65%	0,70%
**GWP**	kg CO2-Eq	1,48E+00	1,35%	68,68%	0,44%	4,56%	3,26%	0,82%	3,00%	6,69%	3,74%	6,96%	0,50%
**FETP**	kg 1,4-DCB-Eq	1,20E-02	1,54%	55,34%	0,53%	5,72%	6,75%	1,69%	5,08%	4,14%	6,53%	11,40%	1,28%
**METP**	kg 1,4-DCB-Eq	1,76E-02	1,50%	53,24%	0,55%	5,82%	6,35%	1,59%	5,16%	4,04%	6,39%	14,11%	1,25%
**TETP**	kg 1,4-DCB-Eq	2,52E+00	0,47%	28,49%	0,70%	7,52%	3,27%	0,84%	6,20%	5,03%	2,67%	44,44%	0,37%
**FFP**	kg oil-Eq	7,69E-01	0,47%	81,16%	0,44%	3,14%	1,35%	0,34%	2,89%	0,82%	3,87%	5,26%	0,26%
**FEP**	kg P-Eq	1,20E-04	2,46%	39,92%	0,56%	6,78%	10,05%	2,52%	5,98%	5,89%	7,76%	13,64%	4,44%
**MEP**	kg N-Eq	4,86E-05	1,36%	28,68%	0,17%	2,18%	32,54%	8,14%	1,95%	16,79%	1,95%	5,27%	0,97%
**HTPc**	kg 1,4-DCB-Eq	1,90E-02	1,26%	44,72%	1,05%	11,64%	6,20%	1,59%	10,46%	4,27%	4,53%	12,59%	1,69%
**HTPnc**	kg 1,4-DCB-Eq	5,73E-01	1,49%	43,78%	0,49%	4,95%	6,80%	1,71%	4,36%	4,24%	5,58%	25,32%	1,28%
**IRP**	kBq Co-60-Eq	1,19E-01	3,13%	20,36%	0,42%	5,96%	10,30%	2,58%	5,12%	6,19%	8,04%	31,27%	6,63%
**LOP**	m2*a crop-Eq	4,21E-02	0,95%	18,40%	0,18%	3,00%	2,35%	0,59%	2,38%	14,27%	5,21%	51,98%	0,69%
**SOP**	kg Cu-Eq	1,93E+00	0,11%	5,79%	0,03%	0,10%	0,00%	0,00%	0,05%	0,00%	0,00%	93,90%	0,02%
**ODP**	kg CFC-11-Eq	1,59E-06	0,25%	23,65%	0,17%	7,47%	12,65%	3,16%	0,94%	31,85%	0,59%	19,08%	0,19%
**PMFP**	kg PM2.5-Eq	7,39E-03	0,14%	14,13%	0,11%	0,77%	0,49%	0,12%	0,60%	0,65%	0,40%	81,99%	0,60%
**HOFP**	kg NOx-Eq	3,23E-03	1,78%	68,10%	0,39%	4,58%	1,84%	0,46%	2,63%	7,71%	4,37%	7,78%	0,36%
**EOFP**	kg NOx-Eq	3,43E-03	1,70%	67,29%	0,39%	4,45%	1,81%	0,45%	2,60%	7,32%	5,48%	8,16%	0,35%
**WCP**	m3	9,53E-01	99,97%	0,01%	0,00%	0,00%	0,00%	0,00%	0,00%	0,00%	0,00%	0,02%	0,00%

5. Intraossesous odontectomy – average case - Percentage contribution of the different procedural steps to the overall environmental impacts

Impact category	Reference unit	Results	Hand washing	Preparation	Anesthesia	Exploration, drilling	Extraction	Curettage	Bone edge smoothing	Hemostasis, closure	Disinfection	Sterilisation	Waste
**TAP**	kg SO2-Eq	2,21E-02	0,14%	14,16%	0,19%	0,80%	0,91%	0,36%	0,42%	0,95%	0,38%	80,99%	0,70%
**GWP**	kg CO2-Eq	1,56E+00	1,29%	65,42%	0,80%	5,48%	3,91%	1,55%	2,85%	7,13%	3,56%	7,53%	0,48%
**FETP**	kg 1,4-DCB-Eq	1,32E-02	1,40%	50,50%	0,86%	7,75%	7,74%	3,05%	4,61%	5,27%	5,93%	11,69%	1,20%
**METP**	kg 1,4-DCB-Eq	1,93E-02	1,37%	48,83%	0,87%	7,73%	7,32%	2,87%	4,70%	5,10%	5,82%	14,20%	1,19%
**TETP**	kg 1,4-DCB-Eq	2,64E+00	0,45%	27,67%	0,92%	7,85%	4,14%	1,48%	5,92%	5,48%	2,55%	43,16%	0,38%
**FFP**	kg oil-Eq	7,98E-01	0,45%	78,20%	0,82%	4,32%	1,64%	0,65%	2,78%	1,11%	3,73%	6,02%	0,28%
**FEP**	kg P-Eq	1,36E-04	2,17%	35,47%	0,84%	8,92%	11,17%	4,40%	5,27%	7,37%	6,84%	13,51%	4,04%
**MEP**	kg N-Eq	6,14E-05	1,08%	22,80%	0,24%	2,40%	32,23%	12,88%	1,54%	19,73%	1,55%	4,75%	0,80%
**HTPc**	kg 1,4-DCB-Eq	2,07E-02	1,15%	41,69%	1,29%	12,49%	7,48%	2,73%	9,57%	5,18%	4,14%	12,68%	1,60%
**HTPnc**	kg 1,4-DCB-Eq	6,26E-01	1,37%	40,34%	0,78%	6,48%	7,86%	3,09%	3,99%	5,41%	5,11%	24,34%	1,23%
**IRP**	kBq Co-60-Eq	1,37E-01	2,73%	18,02%	0,65%	8,73%	11,29%	4,48%	4,47%	7,63%	7,02%	29,01%	5,97%
**LOP**	m2*a crop-Eq	5,10E-02	0,78%	18,71%	0,28%	2,85%	2,44%	0,97%	1,96%	12,26%	4,30%	54,86%	0,59%
**SOP**	kg Cu-Eq	1,93E+00	0,11%	5,77%	0,06%	0,19%	0,00%	0,00%	0,05%	0,00%	0,00%	93,79%	0,03%
**ODP**	kg CFC-11-Eq	1,75E-06	0,23%	21,48%	0,31%	7,02%	14,35%	5,73%	0,85%	31,74%	0,54%	17,58%	0,17%
**PMFP**	kg PM2.5-Eq	7,47E-03	0,14%	14,02%	0,19%	0,98%	0,61%	0,24%	0,59%	0,76%	0,40%	81,47%	0,60%
**HOFP**	kg NOx-Eq	3,35E-03	1,72%	65,87%	0,72%	5,16%	2,25%	0,88%	2,54%	7,88%	4,21%	8,43%	0,34%
**EOFP**	kg NOx-Eq	3,55E-03	1,64%	65,09%	0,72%	5,04%	2,21%	0,87%	2,51%	7,50%	5,29%	8,79%	0,34%
**WCP**	m3	9,53E-01	99,97%	0,01%	0,00%	0,00%	0,00%	0,00%	0,00%	0,01%	0,00%	0,01%	0,00%

6. Intraosseous odontectomy – worst case - Percentage contribution of the different procedural steps to the overall environmental impacts

Impact category	Reference unit	Results	Hand washing	Preparation	Anesthesia	Exploration, drilling	Extraction	Curettage	Bone edge smoothing	Hemostasis, closure	Disinfection	Sterilisation	Waste
**TAP**	kg SO2-Eq	3,22E-02	0,10%	16,46%	0,25%	1,07%	23,26%	0,37%	0,29%	1,04%	0,39%	56,02%	0,75%
**GWP**	kg CO2-Eq	2,35E+00	0,85%	66,29%	1,04%	7,00%	3,30%	1,53%	1,89%	8,40%	3,18%	6,03%	0,49%
**FETP**	kg 1,4-DCB-Eq	1,98E-02	0,93%	54,03%	1,58%	9,55%	6,46%	3,03%	3,07%	4,89%	5,90%	9,34%	1,22%
**METP**	kg 1,4-DCB-Eq	2,90E-02	0,91%	52,28%	1,52%	9,54%	6,88%	2,85%	3,13%	4,77%	5,89%	11,01%	1,21%
**TETP**	kg 1,4-DCB-Eq	3,94E+00	0,30%	29,14%	1,07%	10,28%	13,95%	1,45%	3,96%	6,02%	3,61%	29,84%	0,38%
**FFP**	kg oil-Eq	1,19E+00	0,30%	80,09%	0,77%	5,14%	1,36%	0,65%	1,87%	1,05%	3,38%	5,11%	0,28%
**FEP**	kg P-Eq	2,01E-04	1,47%	38,81%	2,05%	11,49%	9,53%	4,46%	3,57%	6,89%	6,60%	10,96%	4,17%
**MEP**	kg N-Eq	9,15E-05	0,72%	23,52%	4,48%	7,07%	26,01%	12,95%	1,03%	17,81%	1,68%	3,91%	0,82%
**HTPc**	kg 1,4-DCB-Eq	3,32E-02	0,72%	48,14%	1,61%	14,93%	7,40%	2,50%	5,98%	4,31%	3,65%	9,23%	1,53%
**HTPnc**	kg 1,4-DCB-Eq	9,35E-01	0,92%	43,49%	1,54%	8,33%	10,52%	3,09%	2,67%	5,07%	5,46%	17,66%	1,25%
**IRP**	kBq Co-60-Eq	1,98E-01	1,88%	20,85%	1,98%	11,10%	13,78%	4,63%	3,08%	7,37%	6,83%	22,19%	6,31%
**LOP**	m2*a crop-Eq	7,60E-02	0,53%	16,60%	0,51%	3,89%	2,04%	0,98%	1,32%	15,80%	5,87%	51,87%	0,59%
**SOP**	kg Cu-Eq	2,80E+00	0,07%	7,96%	0,04%	0,20%	26,55%	0,00%	0,04%	0,00%	0,00%	65,11%	0,03%
**ODP**	kg CFC-11-Eq	3,09E-06	0,13%	26,17%	1,80%	9,43%	13,37%	4,87%	0,48%	32,68%	0,68%	10,24%	0,15%
**PMFP**	kg PM2.5-Eq	1,09E-02	0,10%	16,45%	0,21%	1,26%	23,12%	0,24%	0,40%	0,86%	0,43%	56,30%	0,63%
**HOFP**	kg NOx-Eq	5,02E-03	1,14%	66,31%	0,77%	6,66%	2,09%	0,88%	1,69%	9,88%	3,51%	6,71%	0,36%
**EOFP**	kg NOx-Eq	5,31E-03	1,10%	65,86%	0,77%	6,51%	2,17%	0,87%	1,68%	9,42%	4,30%	6,97%	0,35%
**WCP**	m3	9,53E-01	99,95%	0,01%	0,00%	0,00%	0,01%	0,00%	0,00%	0,01%	0,00%	0,02%	0,00%

Uncertainity analysis:

1. Submucosus odontectomy- ideal case

Impact category	Reference unit	Mean	Standard deviation	Minimum	Maximum	Median	5% Percentile	95% Percentile
**TAP**	kg SO2-Eq	2,16E-02	5,97E-04	2,04E-02	2,47E-02	2,15E-02	2,08E-02	2,27E-02
**GWP**	kg CO2-Eq	1,38E+00	3,80E-01	7,11E-01	3,76E+00	1,30E+00	9,14E-01	2,11E+00
**FETP**	kg 1,4-DCB-Eq	1,05E-02	9,27E-03	2,70E-03	1,70E-01	7,94E-03	4,15E-03	2,42E-02
**METP**	kg 1,4-DCB-Eq	1,54E-02	1,31E-02	4,48E-03	2,44E-01	1,19E-02	6,52E-03	3,47E-02
**TETP**	kg 1,4-DCB-Eq	2,19E+00	9,70E-01	1,54E+00	2,73E+01	2,02E+00	1,68E+00	3,18E+00
**FFP**	kg oil-Eq	7,36E-01	3,09E-01	2,70E-01	2,50E+00	6,60E-01	3,96E-01	1,36E+00
**FEP**	kg P-Eq	1,04E-04	5,65E-05	3,12E-05	8,15E-04	9,04E-05	5,04E-05	1,87E-04
**MEP**	kg N-Eq	4,22E-05	8,23E-06	2,43E-05	8,44E-05	4,11E-05	3,09E-05	5,78E-05
**HTPc**	kg 1,4-DCB-Eq	1,41E-02	9,49E-03	6,10E-03	1,55E-01	1,17E-02	7,93E-03	2,59E-02
**HTPnc**	kg 1,4-DCB-Eq	5,05E-01	3,92E-01	1,93E-01	5,81E+00	3,95E-01	2,47E-01	1,14E+00
**IRP**	kBq Co-60-Eq	1,02E-01	8,42E-02	2,99E-02	9,79E-01	7,48E-02	3,94E-02	2,44E-01
**LOP**	m2*a crop-Eq	3,88E-02	7,15E-03	2,32E-02	8,67E-02	3,80E-02	2,86E-02	5,10E-02
**SOP**	kg Cu-Eq	1,92E+00	4,42E-04	1,92E+00	1,92E+00	1,92E+00	1,92E+00	1,92E+00
**ODP**	kg CFC-11-Eq	1,50E-06	1,63E-07	1,06E-06	2,26E-06	1,49E-06	1,26E-06	1,79E-06
**PMFP**	kg PM2.5-Eq	7,28E-03	1,98E-04	6,85E-03	8,42E-03	7,24E-03	7,02E-03	7,64E-03
**HOFP**	kg NOx-Eq	3,03E-03	6,84E-04	1,63E-03	6,47E-03	2,90E-03	2,19E-03	4,37E-03
**EOFP**	kg NOx-Eq	3,21E-03	7,02E-04	1,74E-03	6,85E-03	3,08E-03	2,35E-03	4,57E-03
**WCP**	m3	9,61E-01	2,19E-01	4,54E-01	1,91E+00	9,42E-01	6,34E-01	1,36E+00

2. Submucosus odontectomy – average case

Impact category	Reference unit	Mean	Standard deviation	Minimum	Maximum	Median	5% Percentile	95% Percentile
**TAP**	kg SO2-Eq	2,18E-02	4,43E-04	2,09E-02	2,38E-02	2,17E-02	2,12E-02	2,26E-02
**GWP**	kg CO2-Eq	1,43E+00	2,97E-01	9,29E-01	3,15E+00	1,38E+00	1,06E+00	1,98E+00
**FETP**	kg 1,4-DCB-Eq	1,15E-02	9,10E-03	3,82E-03	1,39E-01	9,02E-03	5,07E-03	2,57E-02
**METP**	kg 1,4-DCB-Eq	1,68E-02	1,28E-02	6,15E-03	1,96E-01	1,34E-02	7,88E-03	3,61E-02
**TETP**	kg 1,4-DCB-Eq	2,27E+00	6,10E-01	1,63E+00	8,21E+00	2,11E+00	1,79E+00	3,33E+00
**FFP**	kg oil-Eq	7,36E-01	2,19E-01	3,31E-01	2,13E+00	6,96E-01	4,53E-01	1,14E+00
**FEP**	kg P-Eq	1,13E-04	4,96E-05	3,54E-05	7,81E-04	1,03E-04	5,86E-05	2,04E-04
**MEP**	kg N-Eq	5,54E-05	1,13E-05	3,34E-05	1,09E-04	5,34E-05	4,01E-05	7,61E-05
**HTPc**	kg 1,4-DCB-Eq	1,58E-02	9,02E-03	7,06E-03	9,19E-02	1,33E-02	9,12E-03	3,03E-02
**HTPnc**	kg 1,4-DCB-Eq	5,50E-01	5,13E-01	2,16E-01	1,00E+01	4,36E-01	2,75E-01	1,11E+00
**IRP**	kBq Co-60-Eq	1,13E-01	1,01E-01	3,07E-02	1,32E+00	8,28E-02	4,17E-02	2,90E-01
**LOP**	m2*a crop-Eq	4,74E-02	9,01E-03	2,98E-02	9,01E-02	4,61E-02	3,47E-02	6,39E-02
**SOP**	kg Cu-Eq	1,93E+00	5,05E-04	1,92E+00	1,93E+00	1,93E+00	1,93E+00	1,93E+00
**ODP**	kg CFC-11-Eq	1,66E-06	1,74E-07	1,23E-06	2,50E-06	1,65E-06	1,42E-06	1,97E-06
**PMFP**	kg PM2.5-Eq	7,33E-03	1,39E-04	7,05E-03	7,93E-03	7,31E-03	7,14E-03	7,61E-03
**HOFP**	kg NOx-Eq	3,12E-03	5,86E-04	2,22E-03	7,60E-03	2,99E-03	2,46E-03	4,13E-03
**EOFP**	kg NOx-Eq	3,31E-03	5,93E-04	2,36E-03	7,83E-03	3,19E-03	2,63E-03	4,33E-03
**WCP**	m3	9,37E-01	2,25E-01	4,22E-01	1,89E+00	9,11E-01	6,15E-01	1,34E+00

3. Submucosus odontectomy – worst case

Impact category	Reference unit	Mean	Standard deviation	Minimum	Maximum	Median	5% Percentile	95% Percentile
**TAP**	kg SO2-Eq	3,19E-02	9,41E-04	3,02E-02	3,71E-02	3,17E-02	3,07E-02	3,36E-02
**GWP**	kg CO2-Eq	2,19E+00	5,85E-01	1,19E+00	6,25E+00	2,07E+00	1,49E+00	3,22E+00
**FETP**	kg 1,4-DCB-Eq	1,74E-02	1,40E-02	4,88E-03	2,63E-01	1,38E-02	7,72E-03	3,91E-02
**METP**	kg 1,4-DCB-Eq	2,54E-02	1,98E-02	7,90E-03	3,76E-01	2,04E-02	1,19E-02	5,61E-02
**TETP**	kg 1,4-DCB-Eq	3,45E+00	9,14E-01	2,36E+00	1,06E+01	3,19E+00	2,67E+00	4,98E+00
**FFP**	kg oil-Eq	1,12E+00	4,21E-01	3,93E-01	3,76E+00	1,03E+00	6,27E-01	1,92E+00
**FEP**	kg P-Eq	1,76E-04	9,25E-05	6,57E-05	1,87E-03	1,56E-04	9,16E-05	3,10E-04
**MEP**	kg N-Eq	8,54E-05	1,82E-05	4,67E-05	1,80E-04	8,30E-05	6,05E-05	1,19E-04
**HTPc**	kg 1,4-DCB-Eq	2,73E-02	1,77E-02	1,12E-02	2,94E-01	2,33E-02	1,49E-02	4,93E-02
**HTPnc**	kg 1,4-DCB-Eq	8,11E-01	6,05E-01	3,41E-01	1,06E+01	6,60E-01	4,27E-01	1,70E+00
**IRP**	kBq Co-60-Eq	1,72E-01	1,70E-01	4,61E-02	2,82E+00	1,25E-01	5,91E-02	4,51E-01
**LOP**	m2*a crop-Eq	7,29E-02	1,37E-02	4,29E-02	1,58E-01	7,10E-02	5,41E-02	9,69E-02
**SOP**	kg Cu-Eq	2,79E+00	5,66E-04	2,79E+00	2,79E+00	2,79E+00	2,79E+00	2,79E+00
**ODP**	kg CFC-11-Eq	3,01E-06	3,17E-07	2,24E-06	4,56E-06	2,99E-06	2,53E-06	3,55E-06
**PMFP**	kg PM2.5-Eq	1,07E-02	3,04E-04	1,01E-02	1,24E-02	1,07E-02	1,04E-02	1,13E-02
**HOFP**	kg NOx-Eq	4,83E-03	1,14E-03	2,96E-03	1,63E-02	4,60E-03	3,46E-03	7,06E-03
**EOFP**	kg NOx-Eq	5,11E-03	1,17E-03	3,17E-03	1,65E-02	4,86E-03	3,69E-03	7,36E-03
**WCP**	m3	9,62E-01	2,13E-01	4,66E-01	1,81E+00	9,40E-01	6,49E-01	1,34E+00

4. Intraosseous odontectomy – ideal case

Impact category	Reference unit	Mean	Standard deviation	Minimum	Maximum	Median	5% Percentile	95% Percentile
**TAP**	kg SO2-Eq	2,19E-02	5,77E-04	2,07E-02	2,53E-02	2,18E-02	2,11E-02	2,29E-02
**GWP**	kg CO2-Eq	1,47E+00	3,73E-01	7,38E-01	4,10E+00	1,41E+00	9,99E-01	2,13E+00
**FETP**	kg 1,4-DCB-Eq	1,22E-02	8,21E-03	3,77E-03	9,15E-02	9,69E-03	5,25E-03	2,77E-02
**METP**	kg 1,4-DCB-Eq	1,79E-02	1,15E-02	5,90E-03	1,29E-01	1,45E-02	8,15E-03	3,95E-02
**TETP**	kg 1,4-DCB-Eq	2,51E+00	5,90E-01	1,71E+00	6,24E+00	2,35E+00	1,90E+00	3,69E+00
**FFP**	kg oil-Eq	7,92E-01	3,02E-01	3,37E-01	4,08E+00	7,29E-01	4,53E-01	1,34E+00
**FEP**	kg P-Eq	1,20E-04	5,28E-05	4,25E-05	5,32E-04	1,07E-04	6,29E-05	2,16E-04
**MEP**	kg N-Eq	4,89E-05	8,69E-06	2,92E-05	8,60E-05	4,76E-05	3,65E-05	6,47E-05
**HTPc**	kg 1,4-DCB-Eq	1,90E-02	1,00E-02	8,53E-03	1,80E-01	1,62E-02	1,13E-02	3,56E-02
**HTPnc**	kg 1,4-DCB-Eq	5,97E-01	5,81E-01	2,13E-01	1,03E+01	4,62E-01	2,91E-01	1,31E+00
**IRP**	kBq Co-60-Eq	1,20E-01	1,11E-01	3,16E-02	1,49E+00	8,57E-02	4,26E-02	3,42E-01
**LOP**	m2*a crop-Eq	4,21E-02	6,96E-03	2,33E-02	7,28E-02	4,16E-02	3,18E-02	5,47E-02
**SOP**	kg Cu-Eq	1,93E+00	4,13E-04	1,92E+00	1,93E+00	1,93E+00	1,92E+00	1,93E+00
**ODP**	kg CFC-11-Eq	1,59E-06	1,70E-07	1,17E-06	2,43E-06	1,58E-06	1,34E-06	1,91E-06
**PMFP**	kg PM2.5-Eq	7,40E-03	1,86E-04	7,00E-03	8,41E-03	7,37E-03	7,14E-03	7,73E-03
**HOFP**	kg NOx-Eq	3,30E-03	7,43E-04	1,95E-03	7,94E-03	3,13E-03	2,40E-03	4,71E-03
**EOFP**	kg NOx-Eq	3,50E-03	7,59E-04	2,08E-03	8,22E-03	3,33E-03	2,58E-03	4,90E-03
**WCP**	m3	9,44E-01	2,25E-01	4,29E-01	1,84E+00	9,14E-01	6,31E-01	1,36E+00

5. Intraosseous odontectomy – average case

Impact category	Reference unit	Mean	Standard deviation	Minimum	Maximum	Median	5% Percentile	95% Percentile
**TAP**	kg SO2-Eq	2,21E-02	4,75E-04	2,11E-02	2,43E-02	2,20E-02	2,15E-02	2,29E-02
**GWP**	kg CO2-Eq	1,56E+00	3,19E-01	1,02E+00	3,56E+00	1,50E+00	1,15E+00	2,11E+00
**FETP**	kg 1,4-DCB-Eq	1,30E-02	8,08E-03	4,14E-03	9,81E-02	1,09E-02	6,22E-03	2,67E-02
**METP**	kg 1,4-DCB-Eq	1,91E-02	1,15E-02	6,63E-03	1,42E-01	1,60E-02	9,59E-03	3,93E-02
**TETP**	kg 1,4-DCB-Eq	2,64E+00	8,46E-01	1,81E+00	1,52E+01	2,44E+00	2,00E+00	3,74E+00
**FFP**	kg oil-Eq	8,01E-01	2,59E-01	3,90E-01	2,63E+00	7,47E-01	4,92E-01	1,27E+00
**FEP**	kg P-Eq	1,41E-04	1,80E-04	4,90E-05	5,53E-03	1,22E-04	7,16E-05	2,45E-04
**MEP**	kg N-Eq	6,17E-05	1,35E-05	3,66E-05	1,57E-04	5,92E-05	4,51E-05	8,75E-05
**HTPc**	kg 1,4-DCB-Eq	2,09E-02	1,31E-02	9,92E-03	2,84E-01	1,77E-02	1,28E-02	3,91E-02
**HTPnc**	kg 1,4-DCB-Eq	6,23E-01	4,34E-01	2,55E-01	5,26E+00	5,03E-01	3,28E-01	1,24E+00
**IRP**	kBq Co-60-Eq	1,41E-01	1,29E-01	3,42E-02	1,68E+00	9,86E-02	4,58E-02	3,63E-01
**LOP**	m2*a crop-Eq	5,12E-02	9,64E-03	3,05E-02	1,01E-01	4,95E-02	3,83E-02	6,84E-02
**SOP**	kg Cu-Eq	1,93E+00	5,18E-04	1,93E+00	1,94E+00	1,93E+00	1,93E+00	1,93E+00
**ODP**	kg CFC-11-Eq	1,76E-06	1,83E-07	1,28E-06	2,72E-06	1,75E-06	1,49E-06	2,08E-06
**PMFP**	kg PM2.5-Eq	7,46E-03	1,48E-04	7,18E-03	8,22E-03	7,44E-03	7,27E-03	7,73E-03
**HOFP**	kg NOx-Eq	3,33E-03	5,50E-04	2,32E-03	6,05E-03	3,23E-03	2,64E-03	4,39E-03
**EOFP**	kg NOx-Eq	3,53E-03	5,57E-04	2,47E-03	6,24E-03	3,44E-03	2,81E-03	4,59E-03
**WCP**	m3	9,52E-01	2,21E-01	3,96E-01	2,16E+00	9,36E-01	6,33E-01	1,34E+00

6. Intraosseous odontectomy – worst case

Impact category	Reference unit	Mean	Standard deviation	Minimum	Maximum	Median	5% Percentile	95% Percentile
**TAP**	kg SO2-Eq	3,22E-02	9,51E-04	3,03E-02	3,71E-02	3,21E-02	3,11E-02	3,40E-02
**GWP**	kg CO2-Eq	2,36E+00	6,16E-01	1,28E+00	6,07E+00	2,24E+00	1,64E+00	3,54E+00
**FETP**	kg 1,4-DCB-Eq	1,95E-02	1,29E-02	6,29E-03	1,18E-01	1,56E-02	8,89E-03	4,46E-02
**METP**	kg 1,4-DCB-Eq	2,86E-02	1,82E-02	9,92E-03	1,68E-01	2,30E-02	1,37E-02	6,33E-02
**TETP**	kg 1,4-DCB-Eq	3,87E+00	8,73E-01	2,63E+00	1,04E+01	3,68E+00	2,95E+00	5,51E+00
**FFP**	kg oil-Eq	1,18E+00	4,50E-01	4,55E-01	4,78E+00	1,08E+00	6,70E-01	1,94E+00
**FEP**	kg P-Eq	1,97E-04	8,98E-05	6,17E-05	1,12E-03	1,77E-04	1,05E-04	3,55E-04
**MEP**	kg N-Eq	9,14E-05	1,77E-05	5,24E-05	2,00E-04	8,90E-05	6,75E-05	1,23E-04
**HTPc**	kg 1,4-DCB-Eq	3,24E-02	1,59E-02	1,58E-02	2,18E-01	2,83E-02	2,00E-02	5,77E-02
**HTPnc**	kg 1,4-DCB-Eq	9,05E-01	5,55E-01	3,55E-01	4,62E+00	7,39E-01	4,65E-01	2,05E+00
**IRP**	kBq Co-60-Eq	2,09E-01	2,31E-01	4,53E-02	3,14E+00	1,43E-01	6,76E-02	5,64E-01
**LOP**	m2*a crop-Eq	7,57E-02	1,25E-02	4,66E-02	1,26E-01	7,40E-02	5,76E-02	9,96E-02
**SOP**	kg Cu-Eq	2,80E+00	5,31E-04	2,80E+00	2,80E+00	2,80E+00	2,80E+00	2,80E+00
**ODP**	kg CFC-11-Eq	3,11E-06	3,32E-07	2,27E-06	4,45E-06	3,08E-06	2,61E-06	3,71E-06
**PMFP**	kg PM2.5-Eq	1,09E-02	3,11E-04	1,03E-02	1,27E-02	1,09E-02	1,05E-02	1,15E-02
**HOFP**	kg NOx-Eq	5,07E-03	1,13E-03	2,97E-03	1,54E-02	4,85E-03	3,76E-03	7,20E-03
**EOFP**	kg NOx-Eq	5,35E-03	1,15E-03	3,17E-03	1,56E-02	5,12E-03	4,02E-03	7,51E-03
**WCP**	m3	9,52E-01	2,15E-01	4,82E-01	1,96E+00	9,32E-01	6,42E-01	1,34E+00
